# Polyphyllin I attenuates the invasion and metastasis via downregulating GRP78 in drug-resistant hepatocellular carcinoma cells

**DOI:** 10.18632/aging.205176

**Published:** 2023-11-06

**Authors:** Haiyan Du, Haochen Wu, Qinyang Kang, Mianmian Liao, Meirong Qin, Ning Chen, Houshuang Huang, Danping Huang, Ping Wang, Guangdong Tong

**Affiliations:** 1 Department of Hepatology, Shenzhen Traditional Chinese Medicine Hospital, The Fourth Clinical Medical College of Guangzhou University of Chinese Medicine, Shenzhen 518000, Guangdong, China; 2 Shenzhen Institute for Drug Control, Shenzhen 518000, Guangdong, China; 3 Department of Integrated Traditional Chinese and Western Medicine, School of Clinical Medicine, Guangdong Pharmaceutical University, Guangzhou 510006, Guangdong, China

**Keywords:** Polyphyllin I, invasion and metastasis, sorafenib-resistant, GRP78, hepatocellular carcinoma

## Abstract

Drug resistance to chemotherapy agents presents a major obstacle to the effective treatment of hepatocellular carcinoma (HCC), a common type of liver cancer. Increasing evidence indicates a link between drug resistance and the recurrence of HCC. Polyphyllin I (PPI), a promising pharmaceutical candidate, has shown potential therapeutic advantages in the treatment of sorafenib-resistant hepatocellular carcinoma (SR-HCC cells). In this study, we sought to investigate the mechanism underlying the inhibitory effect of PPI on the invasion and metastasis of SR-HCC cells. Our *in vitro* studies included scratch wound-healing migration assays and transwell assays to examine PPI's effect on HCC cell migration and invasion. Flow cytometry was employed to analyze the accumulation or efflux of chemotherapy drugs. The results of these experiments demonstrated that PPI increased the susceptibility of HCC to sorafenib while inhibiting SR-HCC cell growth, migration, and invasion. Molecular docking analysis revealed that PPI exhibited a higher binding affinity with GRP78. Western blot analysis and immunofluorescence experiments showed that PPI reduced the expression of GRP78, E-cadherin, N-cadherin, Vimentin, and ABCG2 in SR-HCC cells.

Interference with and overproduction of GRP78 *in vitro* impacted the proliferation, migration, invasion, and metastasis of HCC cells. Further examination revealed that PPI hindered the expression of GRP78 protein, resulting in a suppressive effect on SR-HCC cell migration and invasion. Histological examination of tumor tissue substantiated that administering PPI via gavage to HepG2/S xenograft nude mice inhibited tumor growth and significantly reduced tumor size, as evidenced by xenograft experiments involving nude mice. Hematoxylin and eosin (HE) staining of tumor tissue specimens, along with immunohistochemistry (IHC), were conducted to evaluate the expression levels of Ki67, GRP78, N-cadherin, Vimentin, and ABCG2. The results indicated that PPI administration decreased the levels of proteins associated with metastasis and markers of drug resistance in tumor tissues, impeding tumor growth and spread. Overall, our findings demonstrated that PPI effectively suppressed the viability, proliferation, invasion, and metastasis of SR-HCC cells both *in vitro* and *in vivo* by modulating GRP78 activity. These findings provide new insights into the mechanism of PPI inhibition of SR-HCC cell invasion and metastasis, highlighting PPI as a potential treatment option for sorafenib-resistant HCC.

## INTRODUCTION

Liver cancer is a prevalent malignancy both in China and globally [1, 2]. It ranks as the second most common cause of cancer-related deaths worldwide [3], leading to the demise of nearly half a million individuals annually. Despite advancements in diagnosis and treatment, the prognosis for patients with advanced hepatocellular carcinoma (HCC) remains dismal. A significant challenge in HCC treatment lies in the emergence of drug resistance, often resulting in tumor recurrence and progression [4, 5].

In recent years, the FDA has granted approval for sorafenib, a kinase inhibitor targeting multiple molecules, for the treatment of advanced HCC [6–8]. It is now understood that sorafenib acts on various tyrosine kinase receptors, including vascular endothelial growth factor receptors (VEGFR) and fibroblast growth factor receptors (FGFRs), while activating AMP-activated protein kinase (AMPK) to inhibit tumor growth [9–11]. However, the emergence of acquired resistance has become a concern, limiting improvements in survival due to low response rates [12, 13]. Sorafenib resistance in HCC cells has been associated with diverse mechanisms, including the activation of alternative signaling pathways that promote cell survival and proliferation in the presence of sorafenib. Another mechanism involves the upregulation of drug efflux transporters, such as ABC transporters, which reduce intracellular sorafenib levels by pumping it out of cells. Additionally, alterations in the tumor microenvironment, such as hypoxia or immune cell presence, can contribute to sorafenib resistance by affecting cellular metabolism and signaling [14]. Reports suggest that GRP78, known to enhance sorafenib resistance, plays a crucial role in HCC [15–17]. The relationship between GRP78 and sorafenib resistance in HCC is complex and not yet fully understood. While some research suggests a potential link between increased GRP78 expression and sorafenib resistance in HCC cells, the precise involvement of GRP78 in sorafenib resistance remains a subject of ongoing investigation.

Chinese herbal medicine has been employed for centuries to treat a wide range of human ailments. Based on prior evidence [18–20]. Polyphyllin I (PPI), a steroidal saponin derived from the rhizome of *Paris polyphylla*, exhibits anti-cancer properties in various cancer types, making it a promising candidate for anti-cancer therapy. While PPI has demonstrated anti-tumor effects in HCC cells, the precise mechanisms underlying these effects are currently under investigation and may involve multiple pathways. Several studies indicate that PPI induces impressive anti-cancer effects by promoting apoptosis in various tumor types, including HCC [21–23]. Some studies have suggested that PPI may inhibit HCC by reducing GRP78 expression [15, 16]. In summary, the exact mechanisms by which PPI exerts its anti-tumor effects in HCC and its relationship with sorafenib resistance are subjects of ongoing research.

While previous studies have demonstrated the anti-cancer properties of PPI, the specific mechanisms responsible for its effects remain largely unexplored. Therefore, the aim of this study was to comprehensively investigate the influence of PPI on GRP78, its correlation with sorafenib resistance in HCC cells, and the potential mechanism underlying the development of drug resistance, invasion, and metastasis in HCC cells. Indeed, the development of novel therapeutic approaches capable of effectively improving clinical outcomes for HCC patients will be of paramount importance.

Despite the well-established effectiveness of PPI in cancer treatment, there remains a gap in our understanding of the mechanisms by which it operates. Consequently, the objective of this investigation was to comprehensively elucidate the impact of PPI on GRP78, its association with sorafenib resistance in HCC cells, and the potential mechanism by which it induces drug resistance, invasion, and metastasis in HCC cells. These findings hold significant importance for advancing novel therapeutic strategies that can substantially enhance clinical outcomes for this patient population.

## MATERIALS AND METHODS

### Materials and reagents

PPI (S9114) and sorafenib (S1040) were purchased from Selleck Chemicals (Selleck, USA). CCK-8 reagent and 4,6-diamidino-2-phenylindole (DAPI) were acquired from Sigma-Aldrich (USA). Fetal bovine serum (FBS) and RPMI 1640 medium were obtained from Gibco (Gibco, USA). Crystal violet was purchased from UCB (Brussels, Belgium). Immunohistochemistry (IHC) was conducted using commercially accessible reagents in accordance with the instructions provided by the manufacturers.

### Cell culture

Human hepatoma cell lines (HepG2 and Huh-7) were obtained from the American Type Culture Collection (ATCC; USA). Sorafenib-resistant human hepatoma cell lines (HepG2/S and Huh-7/S) were purchased from KeyGEN Biotechnology Company (Nanjing, China). Cells were cultured in RPMI 1640 medium containing 10% FBS and 1% penicillin/streptomycin at 37°C and 5% CO_2_.

### Cell counting Kit-8 (CCK-8) assay

A CCK-8 assay was conducted to confirm the viability of cells after each experimental treatment. Inoculations of 3 × 10^3^ cells per well were made in 96-well plates during the logarithmic growth phase. Once the cells had adhered to the wells, they were divided into different groups and treated with various concentrations of PPI for various durations, as indicated. Following the treatment, 10 μL of CCK-8 reagent was introduced into every well and incubated at a temperature of 37°C for a duration of 4 h. The optical density (OD) at 450 nm for each well was measured using microplate readers.

### Assessment of cell viability

The viability of cells was evaluated by staining with Hoechst 33342, which does not require fixation and specifically stains live cells. After discarding the media from the culture, the cell/collagen mixture was washed twice with PBS and then incubated with Hoechst 33342 (2.5 μg/mL in PBS) at 37°C for 30 min. After being washed three times in PBS, Hoechst 33342-stained cells were visualized using a DAPI filter with an Olympus I × 71 fluorescent microscope, 10× magnification, and a DP70 digital camera. To perform cell counting, a total of 300,000 cells were placed in every well of a 6-well plate and given time to adhere to the plate overnight. Following administering the suitable therapeutic medications, the count of cells was assessed using trypan blue exclusion on a Cellometer Mini device (Nexcelom, USA).

### Clonogenic assay

To evaluate HCC cell’s colony-forming capacity, plate clone formation assays were performed. A total of 500 cells were added to 2 mL of 10% 1640 medium in every well of a six-well plate. The plate was then placed in a 37°C incubator with CO_2_ for a duration of 3 days. Afterward, the cells were exposed to different levels of PPI for a duration of 12 days until observable clusters emerged. The colonies were rinsed with PBS and then treated with 4% PFA for 30 minutes prior to being stained using 0.5% crystal violet. Stained colonies were photographed and counted.

### Analysis of flow cytometry

For the drug efflux assay, 3 × 10^5^ cells were seeded into each well of a 6-well plate and allowed to attach before being pretreated with 2.5 μM PPI formulation. The PPI-containing medium was replaced with a new complete medium containing 10 μg/mL sorafenib (Selleck, Shanghai, China) and then incubated at 37°C for 24 h. Next, the cells were rinsed and collected using PBS for flow cytometry examination employing a BD LSR Fortessa (BD Biosciences, USA). FlowJo cytometry analysis software was utilized for the analysis of the data.

### Wound healing migration assay

A wound-healing migration assay was conducted to evaluate the migration ability of SR-HCC cells. Once the cells reached 90–100% confluence in 6-well plates, cells were subjected to serum-free medium for 12 h. Following serum deprivation, two lines were scraped within the SR-HCC cells using a sterile plastic pipette tip in each cultured well, and they were exposed to a serum-free medium for 12 h. The cells were then washed three times with 1 × PBS to remove cellular debris and treated with various concentrations of PPI for 48 h. After 0, 24, and 48 h, the cells were observed under an inverted microscope. The evaluation of cell migration ability involved the calculation of the wound-healing rate percentage (migration distance divided by original wound distance, multiplied by 100%).

### Transwell invasion assay

The transwell invasion assay was employed to detect the cell invasion ability. The cells were placed in the transwell chamber after being suspended in a serum-free 1640 medium. The chamber held a combined capacity of 200 μL of liquid, with an additional 550 μL of complete medium added to the exterior of the chamber. After an overnight incubation, the cells were subsequently exposed to varying amounts of PPI for a duration of 48 h. The medium was removed, and the cells were rinsed thrice with PBS and then treated with 4% paraformaldehyde for 1 h. Subsequently, the cells were dyed with 0.1% crystal violet for 30 min, washed thrice with PBS, and examined using a microscope.

### Molecular docking studies

The process of molecular docking was carried out using Discovery Studio 3.5 and Autodock Vina software. The crystalline structures of proteins (GRP78 for PPI) were obtained by eliminating unwanted co-crystallized ligands and water molecules with the assistance of Discovery Studio Visualizer (Accelrys, USA). AutoDock Tools were utilized to configure input pdbqt files for the protein and ligand, as well as establish the dimensions and central position of the grid box. Using a scoring function, the docking grid was used to dock the ligand conformations. The analysis of ligand-protein binding and interactions was performed using Discovery Studio Visualizer. BC-1480 was used as a framework for synthesizing other compounds by utilizing docking and analysis to determine the most suitable ligands.

### Analysis of Western blot

The cells were lysed on ice by utilizing RIPA lysis buffer (KGP702, KeyGEN Biotechnology Co., Ltd., China) with the addition of 1% PMSF (KGP61, KeyGEN Biotechnology Co., Ltd.). Subsequently, the protein concentration of the lysates was assessed. SDS-PAGE was used to separate the proteins. After separation, the proteins were moved to a PVDF membrane (C55008, Millipore, USA) and obstructed with skimmed milk in TBS-0.05% for 1 h. Subsequently, they were incubated overnight at 4°C with shaking in primary antibodies diluted 1:1000 in Antibody Diluent Solution (00-3218, Invitrogen, USA). At 4°C, the membranes were incubated overnight with β-actin antibody (#3700S, CST, USA), GRP78 antibody (#3177, 1:1000; CST), E-cadherin (#14472, 1:1000; CST), N-cadherin (#13116, 1:1000; CST), ABCG2 antibody (#42078, 1:1000; CST), and Vimentin (#5741, 1:1000; CST). After being washed in TBST, the membranes were incubated with horseradish peroxidase-conjugated secondary antibodies. These antibodies included Goat anti-mouse IgG (AS003, Abclonal, USA, 1:10000) or goat anti-rabbit IgG (AS014, Abclonal, 1:10000), and the incubation took place for 1 h at room temperature. Following that, the membranes were rinsed four times for 5 min each with TBST. The Bio-Rad gel imaging system was utilized to detect bands through chemiluminescence reaction and film exposure, while Western blots were quantified using ImageJ software.

### Immunofluorescence staining

The cells were cultured on chamber slides and treated with PPI (1.5 μM and 3 μM) for 48 h once adherent. Cells were washed with PBS three times and fixed with 4% paraformaldehyde for 30 min. PBS was used to wash the cells, followed by 1% Triton-100 for 30 minutes. For blocking, cells were blocked with 5% bovine serum albumin (BSA)/PBS) for 1 h. The cells were then incubated with primary antibodies overnight at 4°C. After washing with PBS, the cells were incubated with Alexa Fluor 488- or 647-conjugated secondary antibodies (CST, USA). DAPI was applied to the nucleus for 15 minutes at room temperature in the dark. Immunofluorescence was detected using a confocal microscope.

### Tumor xenografts in nude mice

HepG2/S cells were grown in RPMI-1640 (Gibco) containing 10% FBS (Sigma) and sorafenib was added to the cell media. Approximately 1 × 10^7^ cells were subcutaneously injected into BALB/C nude mice sourced from the Guangdong Provincial Animal Testing Centre (Guangzhou, China). The tumor volume was calculated using the formula: V (mm^3^) = length (mm) × width (mm)^2^/2. Four weeks after the cells were injected, three groups of mice were selected (3 mice per group). During the modeling period, mice received 0.2 mL of saline solution by gavage once every day. In addition to the 10 mg/mL dose, two additional groups were given a 2.5 and 5.0 mg/kg/d dose. The mice were killed approximately one month after administration. The tumors were weighed and photographed, and tissues were either fixed with 4% paraformaldehyde or frozen in liquid nitrogen and stored at −80°C.

### IHC staining

Tumor tissue excised from mice was fixed in 4% paraformaldehyde and embedded in paraffin. The paraffin-embedded sections were cut into 5 μm thick sections, dewaxed, and rehydrated. Antigen retrieval was performed using a citrate buffer of 0.01 mmol/L at 95°C for 30 min. Next, the sections were then incubated overnight at 4°C with antibodies specific to Ki67 (#2586, 1:10000, CST), GRP78, Vimentin, N-cadherin, and ABCG2. Following the washing process, the sections were incubated with goat anti-rabbit IgG (ab6795, Abcam, UK). The sections were developed using horseradish peroxidase (HRP)-conjugated avidin and 3,3-diaminobenzidine (DAB)-hydrogen peroxide as substrate, followed by the application of hematoxylin to the slides. Photomicrographs were analyzed and quantified using Image Pro Plus (ImageJ).

### HE staining

Standard protocols were employed for HE staining. Following deparaffinization and rehydration in Clearene, the tissue sections underwent staining with hematoxylin and eosin. Subsequently, they were dehydrated through a series of graded alcohol solutions and cleared in xylene. Finally, the prepared slides were mounted, and observations and photographs were taken using an Olympus BX53 microscope.

### Statistical analysis

The statistical analysis was performed using IBM SPSS 21.0 (IBM). Prior to analysis, all data were assessed to ensure normal distribution and homogeneity of variance. To compare characteristics across multiple groups, a one-way analysis of variance (ANOVA) was employed. Significance was considered with a *P*-value less than 0.05.

### Data availability statement

The datasets used and/or analyzed during the current study are available from the corresponding author on reasonable request.

## RESULTS

### Establishment of sorafenib-resistant cell lines

**Figure 1:**
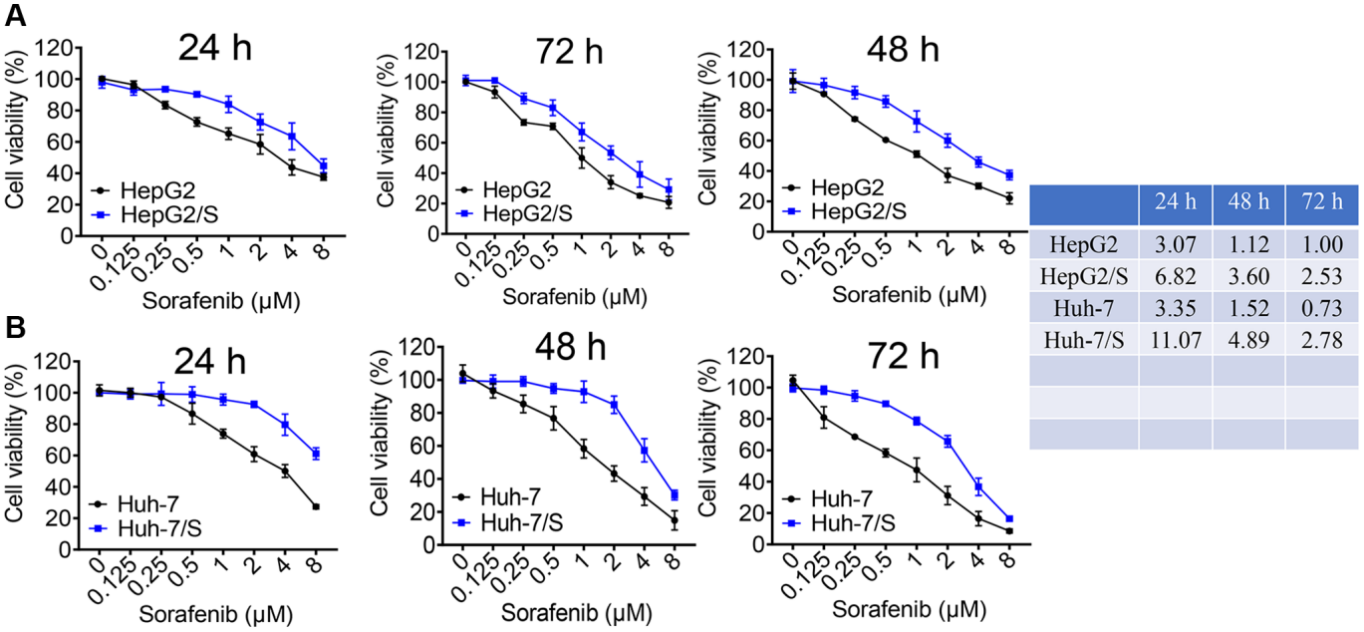
**Identification of sorafenib-resistant cells.** (**A**) The viability of HepG2 and HepG2/S cells was assessed upon treatment with sorafenib at concentrations ranging from 0 to 8 μM. (**B**) The inhibition rate of Sora on Huh-7 and Huh-7/S cells was measured at concentrations ranging from 0 to 8 μM. The IC_50_ value of Sora was determined for HCC/S and HCC cells. A significant increase in the IC_50_ value of cells treated with sorafenib indicates drug resistance compared to the control group.

To investigate the mechanisms underlying sorafenib resistance in HCC, we developed SR-HCC cell lines *in vitro*. Resistance was induced by gradually increasing the sorafenib concentration through repeated passages. Resistant HepG2 and Huh-7 cells were successfully established. A cell viability assay was conducted to determine the 50% inhibitory concentration (IC_50_) of sorafenib in Huh-7/S and HepG2/S cells, as well as their parental counterparts. Additionally, we assessed cellular viability in both sensitive and resistant cells treated with various concentrations of sorafenib for 24, 48, and 72 hours in HCC and SR-HCC cells. The IC_50_ values of HepG2/S cells for sorafenib were 2-3 times higher than those of the parental cells, measuring 6.82 μM, 3.60 μM, and 2.53 μM for 24, 48, and 72 hours, respectively. In contrast, the IC_50_ values for HepG2 cells were 3.07 μM, 1.12 μM, and 1.00 μM for the same time intervals (Figure 1A). Similarly, the IC_50_ values of Huh-7/S cells for sorafenib were 11.05 μM, 4.89 μM, and 2.78 μM, while the IC_50_ values for the parental Huh-7 cells were 3.35 μM, 1.52 μM, and 0.73 μM for 24, 48, and 72 hours, respectively (Figure 1B), consistent with the literature [24], with all resistant cell lines displaying higher IC_50_ values compared to their parental counterparts (Figure 1).

### Effect of PPI on sorafenib-resistant HCC cell proliferation

**Figure 2:**
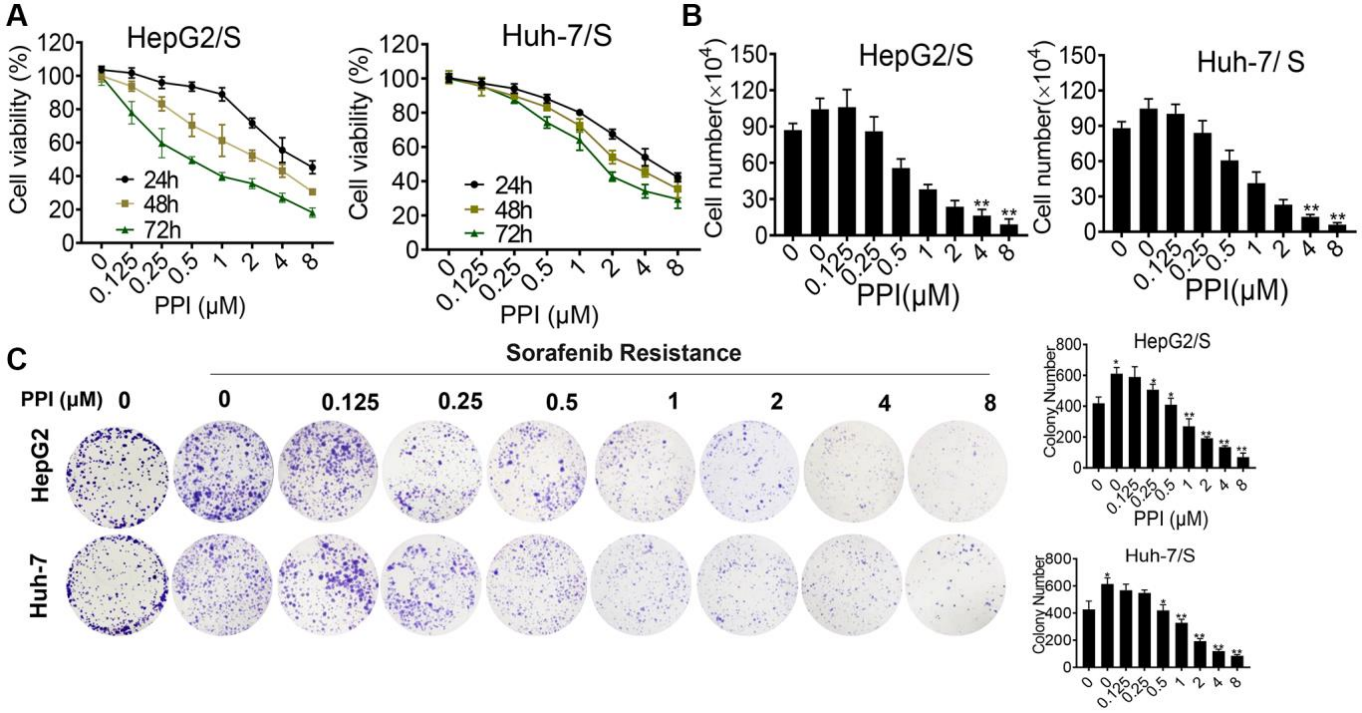
**Cytotoxicity of PPI on HepG2/S and Huh-7/S cells.** (**A**) HepG2/S and Huh-7/S cells were treated with various concentrations of PPI (ranging from 0 to 8 μM) for 24, 48, and 72 h. Cell viability was assessed using the CCK8 assay. The data are presented as means ± SD of three independent experiments. Statistical analysis was performed, and *P*-values less than 0.05 and less than 0.01 were considered significant when compared to control cells. (**B**) The effect of PPI (ranging from 0 to 8 μM) on SR-HCC cell viability was evaluated by cell counting assay after exposing the cells for 24, 48, and 72 h. (**C**) Clone formation assays were performed to examine cell vitality after PPI treatment in SR-HCC. The inhibitory effect of PPI on colony formation was evaluated by treating SR-HCC cells with various concentrations of PPI (ranging from 0 to 8 μM) for 2 weeks, while HCC cells were treated without PPI. Surviving colonies with more than 10 cells were counted. Bar graphs were used to represent the number of clones formed by HCC and SR-HCC cells after various treatments, with statistical significance indicated by asterisks (^*^P < 0.05, ^**^P < 0.01, ^***^P < 0.001).

To evaluate the impact of PPI on the proliferation of SR-HCC cells, we conducted CCK8 assays and colony formation experiments. Absorbance measurements were taken at various time points and concentrations to assess proliferation inhibition. The effects of PPI alone on cell growth and overall cellular toxicity were assessed in SR-HCC cells treated with different PPI concentrations for 24, 48, and 72 hours. The CCK-8 results revealed that PPI strongly inhibited the growth of HepG2/S and Huh-7/S cells, as shown in Figure 2A. Moreover, cell counting assays demonstrated a significant reduction in SR-HCC cell proliferation following PPI treatment, compared to the HCC and SR-HCC cells groups (Figure 2B). In summary, the inhibitory effect of PPI on the proliferation of sorafenib-resistant liver cancer cells was demonstrated through CCK-8 assays, cell counting experiments, and colony formation assays. To investigate the mechanisms underlying PPI's regulatory effect on SR-HCC cells proliferation, colony formation experiments were conducted on SR-HCC cells exposed to varying PPI concentrations, with results indicating a significant inhibition of clonogenic potential as PPI concentration increased, as shown in Figure 2C.

### PPI enhances susceptibility and inhibits invasion and metastasis of sorafenib-resistant liver cancer cells

**Figure 3:**
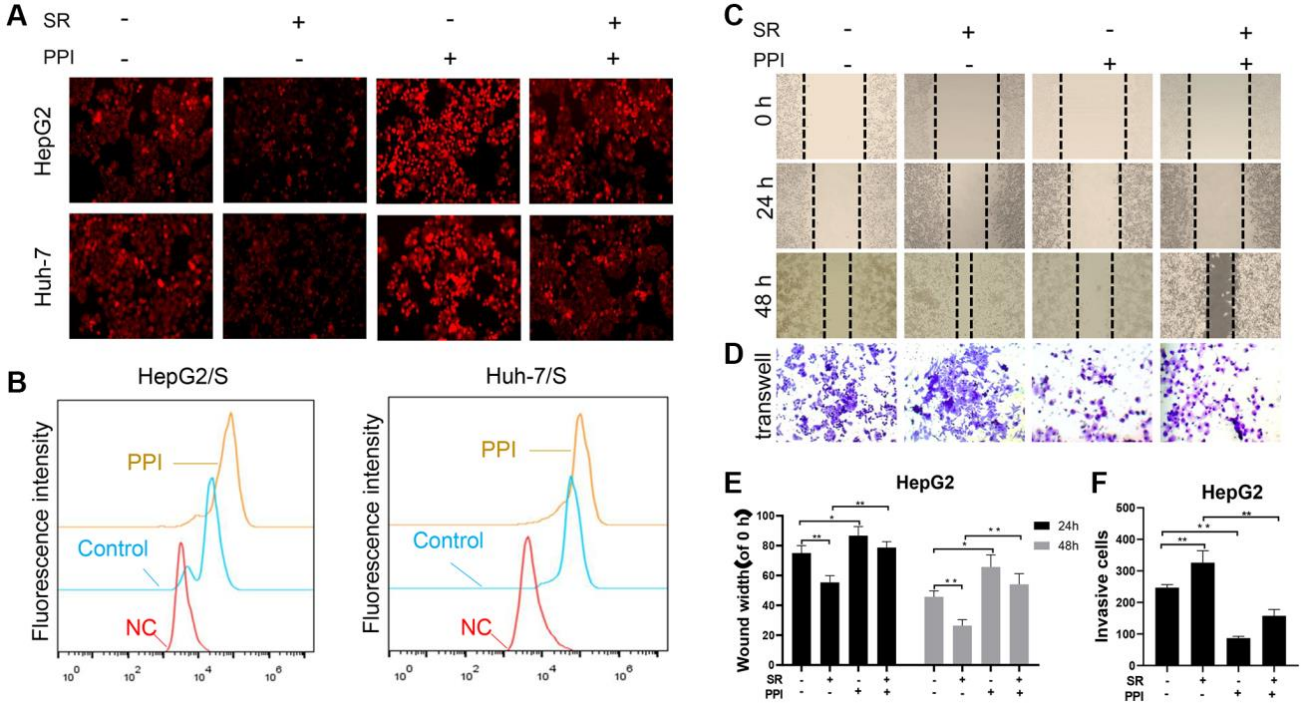
**PPI increases the sensitivity of liver cancer-resistant cells to sorafenib and inhibits their invasion and metastasis.** (**A**, **B**) To measure chemotherapy efflux using flow cytometry, cells are first loaded with a fluorescently labeled chemotherapy drug sorafenib. The HCC cells were then analyzed by flow cytometry to measure the intracellular fluorescence intensity of the PPI or Sora treatment. Intracellular fluorescence intensity was measured using flow cytometry, and the efflux of chemotherapy drugs in cells was quantified and presented in the bar chart. Statistical significance is indicated by asterisks (^*^P < 0.05, ^**^P < 0.01, ^***^P < 0.001). (**C**, **D**) The effects of PPI or Sora on the migration and invasion ability of liver cancer cells. (**E**, **F**) The bar graphs in panels C and D represent the mean ± standard error. ^*^p < 0.05, ^**^p < 0.01.

A drug efflux assay demonstrated a rapid decrease in sorafenib intake in PPI-treated HCC/S cells, evidenced by reduced fluorescence intensities (Figure 3A). This finding was further corroborated by flow cytometry experiments (Figure 3B), collectively suggesting that non-cytotoxic doses of PPI effectively inhibit SR-HCC cells and enhance their chemosensitivity when co-administered with sorafenib. To investigate whether PPI directly impacts SR-HCC cell migration *in vitro*, scratch-wound healing and transwell assays were conducted on HCC cells. Scratch assays revealed migration capacity differences, with control and SR-HCC-treated cells achieving complete wound healing after 48 hours, while PPI-treated and PPI combined with sorafenib-treated cells did not fully heal within the same timeframe, especially in the PPI group. The scratch assay results primarily reflected cell migration abilities, with the PPI group demonstrating incomplete wound healing, indicating that PPI could suppress SR-HCC cell motility without significantly affecting cell proliferation (Figure 3C, 3E). Transwell invasion assays showed that co-administration of PPI and sorafenib significantly inhibited the invasion capacity of SR-HCC cells (Figure 3D, 3F), as evidenced by the reduced abundance of invasive cells following PPI and PPI with sorafenib treatments.

### GRP78 is a key target that promotes the invasion and metastasis of sorafenib-resistant liver cancer cells

**Figure 4:**
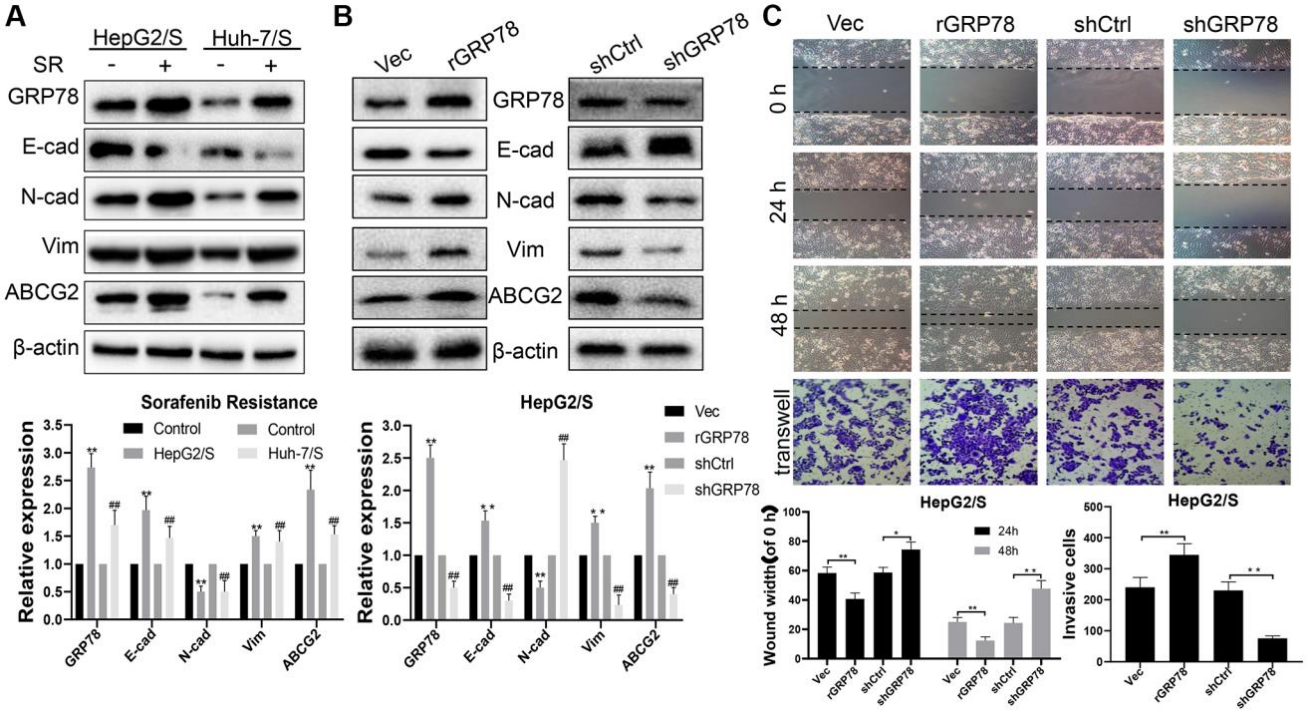
**GRP78 is a key target that increases sorafenib resistance and promotes invasion and metastasis in liver cancer.** (**A**) The effect of sorafenib on the expression of proteins in SR-HCC. The expression levels of proteins (E-cadherin, N-cadherin, Vimentin, ABCG2 and GRP78) in HCC cells treated with sorafenib for 24 h. (**B**) The effects of GRP78 overexpression/knockdown on protein expression in HepG/S cells. (**C**) Effects of GRP78 overexpression/knockdown on migration and invasion of SR-HCC cells.

To investigate the essential role of GRP78 in mediating invasion, metastasis, and drug resistance in SR-HCC cells, we generated stable SR-HCC cell lines with either high or low GRP78 expression through lentiviral vector transfection. Western blot analysis revealed that the presence of sorafenib in HepG2 and Huh-7 cells led to increased expression of GRP78, N-cadherin, Vimentin, and ABCG2, along with decreased levels of E-cadherin, compared to the control group (Figure 4A). HepG2/ S cells with high GRP78 levels exhibited enhanced expression of GRP78, N-cadherin, Vimentin, and ABCG2, and reduced levels of E-cadherin (Figure 4B). Additionally, scratch wound-healing and transwell migration assays demonstrated that overexpression of GRP78 promoted wound healing and facilitated cell invasion in HepG2/S cells (Figure 4C). Conversely, when GRP78 was knocked down, the opposite results were observed, with reduced expression of GRP78, N-cadherin, Vimentin, and ABCG2, and increased E-cadherin levels. Moreover, cell migration and invasion abilities were significantly inhibited in GRP78-interfered SR-HCC cells compared to the Vec group. These findings suggest that the expression level of GRP78 may be a critical factor affecting the therapeutic effectiveness of sorafenib. Furthermore, the correlation between GRP78 and cell invasion and metastasis highlight the significant role of GRP78 in tumorigenesis and disease progression. In summary, the expression of GRP78 protein could influence the resistance of cancer cells to sorafenib, possibly by impacting their invasion and metastasis capabilities.

### PPI Blocks invasion and metastasis of SR-HCC cells by suppressing GRP78

**Figure 5:**
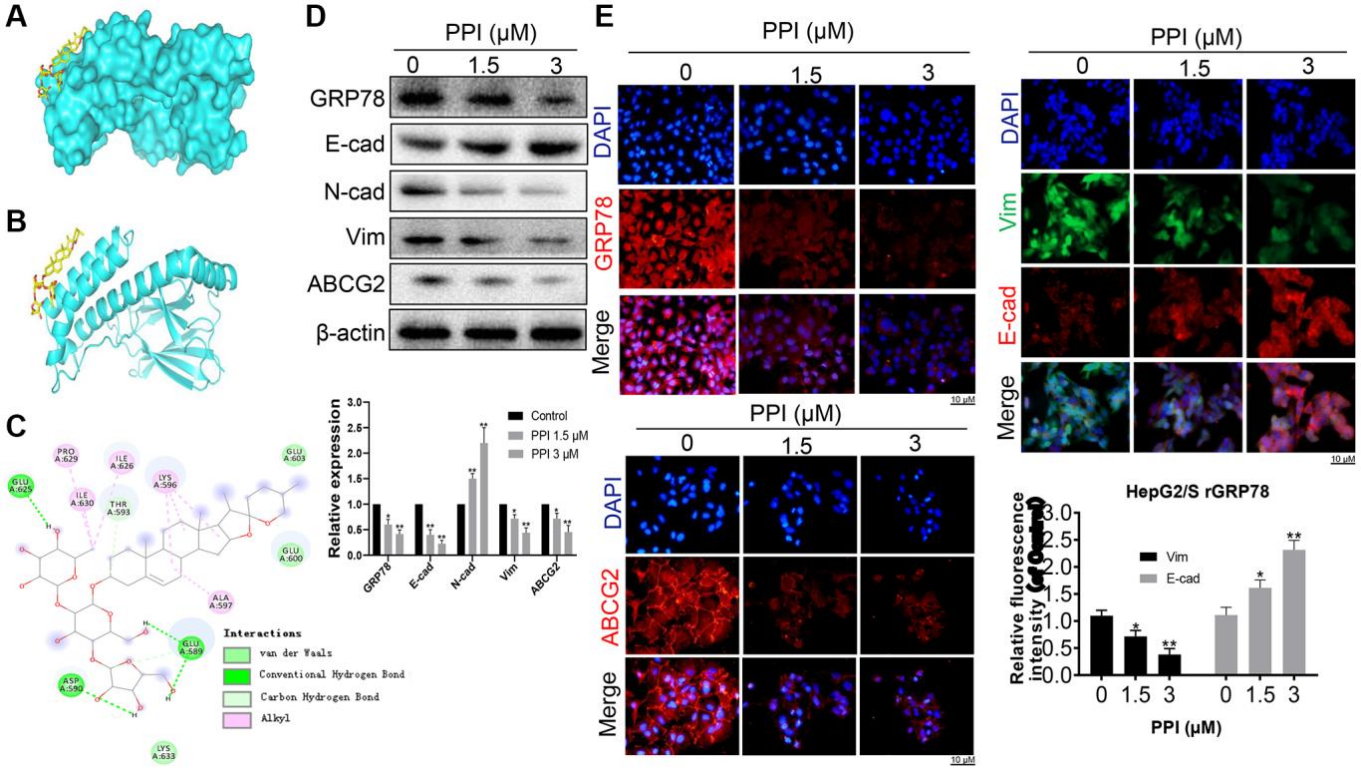
**PPI exerts a blockade on the invasion and metastasis of SR-HCC cells by suppressing GRP78.** (**A**) An ensemble of PPI monomer structures was determined in molecular docking calculations. (**B**, **C**) The results obtained from molecular docking software (AutoDock Vina) are presented. (**D**) Western blot analysis was performed to detect the expression of various proteins in HepG2/S rGRP78 cells. (**E**) Immunofluorescence experiments were conducted to examine the expression of GRP78, E-cadherin, Vimentin, and ABCG2 in HepG2/S rGRP78 cells that had been treated with PPI. Statistical significance is indicated by asterisks (^*^P < 0.05, ^**^P < 0.01, ^***^P < 0.001).

Next, we explored the molecular mechanisms underlying the invasion and metastasis-inhibiting effects of PPI on SR-HCC cells. We performed molecular docking studies using the homology model 3LDP to investigate the binding mode of PPI with GRP78 at the molecular level. The results consistently indicated stable binding of PPI to the ATPase domain of GRP78, characterized by a binding energy of −5.93 kcal/mol (Figure 5A). These findings provide additional evidence supporting the association between PPI and GRP78. Detailed three-dimensional binding poses, binding cavities, and two-dimensional interaction diagrams of the docked complexes are depicted in Figure 5B, 5C.

Subsequently, we assessed the impact of PPI on GRP78 expression in SR-HCC cells. Western blot analysis demonstrated that PPI treatment led to the downregulation of GRP78, N-cadherin, Vimentin, and ABCG2 while upregulating E-cadherin expression (Figure 5D). These observations strongly suggest a positive correlation between GRP78 expression and the levels of invasion and metastasis markers. Moreover, we investigated whether PPI could enhance the sensitivity of liver cancer cells to sorafenib. Sorafenib-resistant HepG2 and Huh-7 cells were exposed to either 1.5 or 3 μM of PPI for 48 hours. The results revealed a dose-dependent reduction in cell viability across all four cell lines treated with PPI (*P* < 0.05), as depicted in Figure 5D. Notably, PPI treatment suppressed the expression of GRP78, N-cadherin and ABCG2 while elevating E-cadherin levels in SR-HCC cells, with significant differences compared to the control group. Besides, PPI treatment yielded a modest inhibitory effect on both sensitive and resistant HCC cells. Immunofluorescence experiments further confirmed significant reductions in the expression of GRP78, N-cadherin, Vimentin, and ABCG2 in HepG2/S cells with depleted rGRP78 (Figure 5E), consistent with the Western blot results. These compelling findings collectively suggest that PPI can restore sensitivity to sorafenib in sorafenib-resistant HCC cells, providing further support for the role of GRP78 in regulating tumor invasion, metastasis, and sorafenib resistance in these cells. In summary, PPI effectively inhibits the invasion and metastasis of sorafenib-resistant liver cancer cells by regulating GRP78 expression in these cells.

### PPI inhibits the proliferation of sorafenib-resistant cells *in vivo*

**Figure 6:**
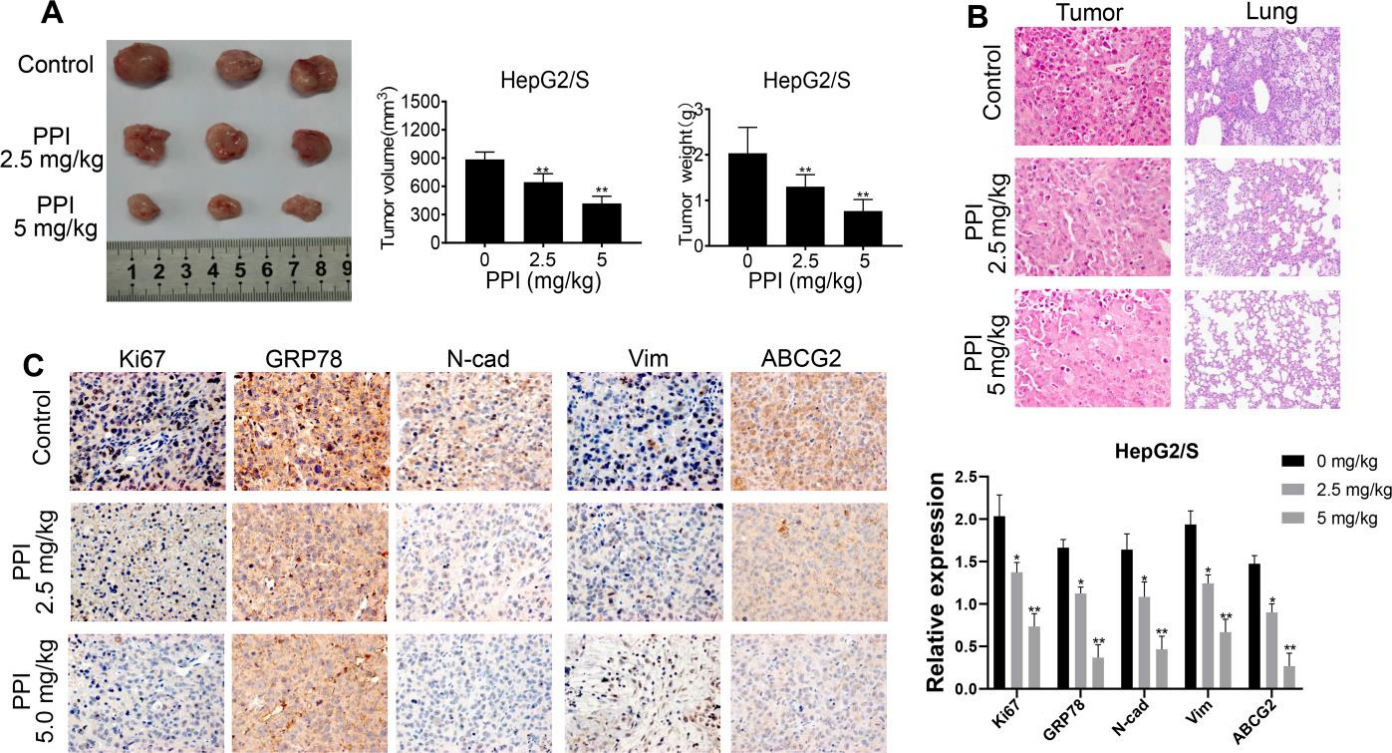
**PPI inhibits the proliferation of sorafenib-resistant cells *in vivo*.** (**A**) PPI treatment inhibited tumor growth and decreased tumor sizes in mice. The tumor weight of mice treated with sorafenib-resistant HepG2 cells with or without PPI administration is represented in a bar graph as mean ± SD. The tumor volume of mice treated with sorafenib-resistant HepG2 cells with or without PPI administration is represented in a bar graph as mean ± SD. (**B**) HE staining of tumor tissues was performed to evaluate the effect of PPI administration. (**C**) IHC analysis was performed for Ki67, GRP78, N-cadherin, Vimentin, and ABCG2 on tumor tissue samples obtained during administration with or without PPI. Statistical significance is indicated by asterisks (^*^P < 0.05, ^**^P < 0.01).

To explore the role of PPI *in vivo*, we conducted experiments using HepG2/S cells injected into Balb/c nude mice. The mice were randomly divided into three groups, and one month after cell injection, they received treatment with PPI (2.5 and 5.0 mg/kg), saline solution, or intragastric administration. After one month of treatment, the mice were euthanized, and tumor volume and weight were measured. The results demonstrated that treatment with PPI at 5.0 mg/kg significantly reduced tumor volume and weight in HepG2/S cells compared to the control group (intragastric administration). Notably, significant differences were observed in mice subjected to PPI administration at a dosage of 2.5 mg/kg, compared to the intragastric administration group, as illustrated in Figure 6A. Subsequent HE staining and immunohistochemistry (IHC) analyses were conducted to examine tumor and lung tissues from HepG2/S xenografts. HE staining results were consistent with the tumor volume findings, with tumor tissue from mice receiving PPI treatment *in vivo* showing a significant reduction compared to the control group. Moreover, the number of lung metastatic nodules was significantly lower in the PPI (2.5 and 5.0 mg/kg) groups than in the control group. Interestingly, the PPI (2.5 mg/kg) group exhibited a higher number of lung metastatic nodules than the PPI (5.0 mg/kg) group (Figure 6B). Immunohistochemistry experiments further demonstrated that PPI-treated groups exhibited significantly decreased expression of Ki67, GRP78, N-cadherin, Vimentin, and ABCG2, consistent with the Western blot results (Figure 6C). Collectively, these findings provide compelling evidence that PPI exerts a significant antitumor effect *in vivo* by impacting the metastasis of HepG2/S cells.

## DISCUSSION

Liver cancer is a major contributor to global cancer-related fatalities, especially in advanced stages with a dismal prognosis. Its high aggressiveness and lethality are often compounded by drug resistance issues [25, 26]. Sorafenib, a multikinase inhibitor, has long been the first-line treatment for advanced liver cancer. Nonetheless, the emergence of sorafenib resistance represents a significant challenge in liver cancer therapy [27]. Therefore, the imperative exists for novel therapeutic strategies capable of overcoming sorafenib resistance and ameliorating the treatment outcomes for patients with advanced liver cancer. One such mechanism driving chemotherapy resistance in cancer cells involves the overexpression of the endoplasmic reticulum chaperone protein GRP78 [28]. PPI, a steroidal saponin derived from *Paris polyphylla*, has exhibited promise as an antitumor agent [29, 30]. Multiple studies substantiated PPI's effectiveness in inhibiting cell proliferation, disrupting the cell cycle, and preventing chemoresistance across various tumor cell lines [31–33]. Notably, no study has hitherto explored the *in vivo* effects of PPI on sorafenib-resistant HCC cells. Our study sought to elucidate PPI's potential to mitigate invasion and metastasis in sorafenib-resistant HCC cells by suppressing GRP78 expression.

Besides, the present study investigated the potential of PPI in overcoming sorafenib resistance while mitigating the invasion and metastasis of liver cancer cells resistant to sorafenib. We employed sorafenib-resistant liver cancer cell lines and exposed them to PPI alone or in conjunction with sorafenib. We assessed cell viability, measured Dox efflux, and conducted assays to evaluate invasion and metastasis. Additionally, we explored the effects of GRP78 overexpression and interference on the invasion and metastasis of sorafenib-resistant liver cancer cells.

Our findings revealed that PPI treatment significantly reduced the invasion and metastasis of drug-resistant HCC cells both *in vitro* and *in vivo*, mediated by the downregulation of GRP78 expression, which subsequently mitigated cancer cell migration and invasion (Figure 4). These results suggest that PPI may hold promise as a treatment for sorafenib-resistant HCC, targeting one of the central mechanisms of chemotherapy resistance. Furthermore, our study sheds light on the molecular mechanisms underpinning PPI's anti-cancer effects, potentially informing the development of novel and more efficacious therapies.

Previous studies have spotlighted the pivotal role of GRP78 in regulating cell survival and resistance to chemotherapy [34, 35]. Our study unveils a critical role for GRP78 in cancer cell invasion and metastasis, adding to its known functions in cell survival and chemotherapy resistance. We observed that PPI-induced downregulation of GRP78 led to a substantial reduction in cancer cell migration and invasion (Figure 5). To validate these findings, we conducted further *in vivo* studies (Figure 6). These results suggest that PPI possesses comprehensive anti-tumor effects that extend beyond inducing cell death. Importantly, our study provided compelling evidence of a direct correlation between PPI, GRP78, and the invasion and dissemination of cancer cells, suggesting that PPI could serve as a potential alternative treatment for individuals with drug-resistant HCC. Additionally, our research highlights the possibility of using the level of GRP78 expression as an indicator for patient selection, with high GRP78 levels potentially signaling a reduced responsiveness to PPI therapy. Nevertheless, the limitations in our study should be acknowledged, including the absence of an examination of the potential side effects of PPI treatment. Additional research is warranted to assess the safety and tolerability of PPI in individuals with cancer.

In addition, we demonstrated that PPI can downregulate the expression of GRP78 in sorafenib-resistant liver cancer cells, which is associated with a significant reduction in cell viability, invasion, and metastasis. These findings suggest that PPI has the potential to overcome sorafenib resistance in liver cancer cells and inhibit cancer cell invasion and metastasis. The downregulation of GRP78 by PPI represents a promising mechanism for overcoming sorafenib resistance and curbing cancer cell invasion and metastasis.

To summarize, our study corroborates that PPI could serve as an effective treatment for sorafenib-resistant HCC by regulating GRP78 expression and diminishing cancer cell invasion and metastasis. These findings carry significant clinical implications and shed light on the molecular mechanisms underlying PPI’s anti-cancer effects. Further studies are warranted to validate our findings and explore the potential of combining PPI with other anti-cancer agents to enhance their therapeutic efficacy.

## CONCLUSIONS

Overall, our findings suggest that PPI treatment leads to a significant reduction in GRP78 expression in sorafenib-resistant HCC cells, consequently enhancing the anti-tumor activity of sorafenib against hepatoma growth, both *in vitro* and *in vivo*. In essence, PPI mitigates the upregulation of GRP78, which may restore cancer cell susceptibility to sorafenib to some extent. These findings provide compelling evidence to support further clinical research into PPI as a potential cancer therapy to augment the effectiveness of sorafenib in treating HCC, especially the sorafenib-resistant variant. However, a notable limitation of our study is that we restricted our investigations to *in vitro* and *in vivo* experiments. Further research is warranted to explore the clinical translation of these findings and comprehensively assess the therapeutic potential of PPI in the context of liver cancer treatment.
